# Expression of trematode-induced zombie-ant behavior is strongly associated with temperature

**DOI:** 10.1093/beheco/arad064

**Published:** 2023-08-24

**Authors:** Simone Nordstrand Gasque, Brian Lund Fredensborg

**Affiliations:** Section for Organismal Biology, Department of Plant and Environmental Sciences, University of Copenhagen, Thorvaldsensvej 40, 1871 Frederiksberg C, Denmark; Section for Organismal Biology, Department of Plant and Environmental Sciences, University of Copenhagen, Thorvaldsensvej 40, 1871 Frederiksberg C, Denmark

**Keywords:** context-dependent behavioral manipulation, *Dicrocoelium dendriticum*, lancet liver fluke, field study, *Formica polyctena*, parasite-induced host behavioral manipulation, temperature

## Abstract

Parasite-induced modification of host behavior increasing transmission to a next host is a common phenomenon. However, field-based studies are rare, and the role of environmental factors in eliciting host behavioral modification is often not considered. We examined the effects of temperature, relative humidity (RH), time of day, date, and an irradiation proxy on behavioral modification of the ant *Formica polyctena* (Förster, 1850) by the brain-encysting lancet liver fluke *Dicrocoelium dendriticum* (Rudolphi, 1819). This fluke induces ants to climb and bite to vegetation by the mandibles in a state of temporary tetany. A total of 1264 individual ants expressing the modified behavior were observed over 13 non-consecutive days during one year in the Bidstrup Forests, Denmark. A sub-set of those ants (*N* = 172) was individually marked to track the attachment and release of infected ants in relation to variation in temperature. Infected ants primarily attached to vegetation early and late in the day, corresponding to low temperature and high RH, presumably coinciding with the grazing activity of potential herbivorous definitive hosts. Temperature was the single most important determinant for the induced phenotypic change. On warm days, infected ants altered between the manipulated and non-manipulated state multiple times, while on cool days, many infected ants remained attached to the vegetation all day. Our results suggest that the temperature sensitivity of the infected ants serves the dual purpose of exposing infected ants to the next host at an opportune time, while protecting them from exposure to high temperatures, which might increase host (and parasite) mortality.

## Teaser

Our study asks whether environmental factors influence behavior change in ants infected with a liver fluke. We investigated the influence of date, time of day, temperature, relative humidity, and a proxy for irradiation. We found that, on warm days, infected ants altered their behavior multiple times, while on cool days, many infected ants remained attached to the vegetation all day. Temperature, therefore, seems to play a meaningful role with respect to inducing behavior modification.

## INTRODUCTION

Parasite manipulation of the host phenotype (appearance and/or behavior) to increase transmission to a new host is a widespread phenomenon across host and parasite phyla (Moore 2002). The phenotypic changes of the infected host may involve the dispersal of infective propagules to favorable locations and at favorable times or, among parasites with a complex life cycle, facilitate an increase in the predation rate of the infected host by the next host in the life cycle of the parasite (i.e., trophic transmission) (Combes 2001; Poulin 2007). Thus, the phenomenon of host phenotypic manipulation is the result of a strong selection pressure on the parasite to enhance the otherwise very small chance of completing its life cycle (Poulin 2007; Froelick et al. 2021). Many cases of host phenotypic manipulation by parasites have been reported (Lafferty and Shaw 2013), but environmental or physiological factors that regulate the expression of the manipulated behavior in infected hosts remain undescribed in most cases (Moore 2002; Lafferty and Shaw 2013; Hughes and Liebersat 2018). Field studies of host behavior in relation to parasite infection are complex and extremely rare since the parasitized host usually displays alterations of already existing behaviors, and parasite contribution to those behaviors may be difficult to quantify under natural conditions (Poulin 1995). The commonality of trophically transmitted parasites in natural ecosystems and their potentially great effect on food web properties (Lafferty et al. 2008) calls for a much better understanding of the environmental factors associated with their transmission. We studied *Dicrocoelium dendriticum*, the lancet liver fluke, which induces a radical but temporary behavioral change in the ant intermediate host, making it easy to phenotypically distinguish infected from uninfected individuals. This quintessential example, therefore, serves as an appropriate model to investigate factors involved in eliciting and maintaining parasite-induced behavioral manipulation under field conditions.

**Figure 1:**
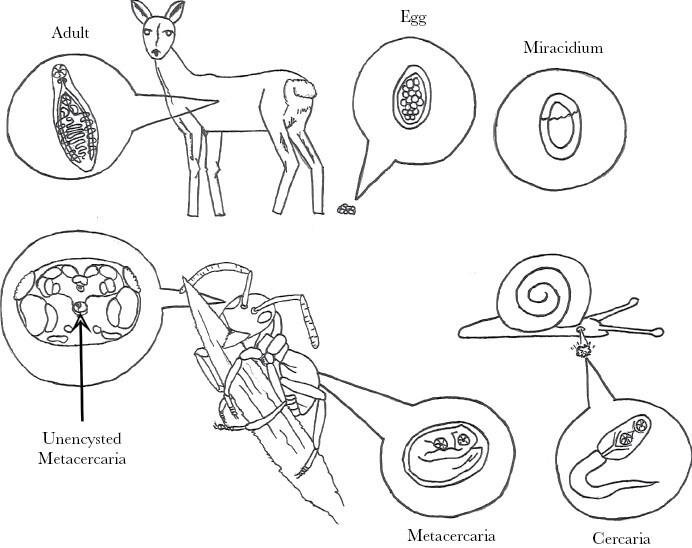
Lifecycle of *Dicrocoelium dendriticum.* Eggs are excreted from an herbivorous mammal serving as the definitive host and hatch into a miracidium when ingested by a terrestrial snail. Mother and daughter sporocysts develop in the hepatopancreas of the snail (not shown), producing the cercaria stage. Cercariae are released from the snail via slimeballs excreted from the pneumastoma. Ants eat the slimeballs containing infective cercariae, which develop into metacercariae in the hemocoel of the gaster of the ant. However, one or more cercariae penetrates the lining of the crop upon ingestion and migrates to the suboesophageal ganglion (depicted in the headcapsule of the ant by arrow; Mart**í**n-Vega et al. 2018), where it induces a reversible biting behavior of the ants where the mandibles are locked to vegetation leaving the ant exposed to ingestion by a suitable definitive host. Following ingestion, the metacercariae excyst in the duodenum, migrate to the bile ducts of the liver and mature to become adult worms producing large numbers of eggs by sexual reproduction. Original drawing by Simone Nordstrand Gasque.

*D. dendriticum* has a complex life cycle, including a terrestrial snail as the first intermediate host, worker ants as the second intermediate host, and herbivorous mammals as the definitive host (Figure 1). *D. dendriticum* relies on trophic transmission for the completion of its life cycle. Individual larval parasites (metacercariae) migrate in an act of altruistic kin selection behavior (Criscione et al. 2020) to the suboesophageal ganglion of the ant (Romig et al. 1980; Mart**í**n-Vega et al. 2018), where they elicit a reversible and radical behavioral change, which is unique to infected hosts (Botnevik et al. 2016). Hence, the infected ant climbs and locks its mandibles to vegetation (Badie and Rondelaud 1988) in a state of tetany that leaves the ant susceptible to ingestion by an herbivorous mammalian definitive host (Tarry 1969). If not ingested by the herbivorous host, the mandibles unlock, and the infected ant is free to return to the forest floor.

In other host–parasite associations, the behavioral change is also radical but precedes the inevitable death of the host (e.g., tree-top disease by baculoviruses and death grip by *Ophiocordyceps*- or *Pandora*-infected ants) (reviewed by Gasque et al. 2019). Thus, in fungal- and viral-induced behaviors where the spores/viral progeny can persist in the environment, the host normally dies within hours/days after the induction of the altered behavior. Contrary to this, *D. dendriticum* cannot survive outside the intermediate ant host, and transmission is dependent on the direct ingestion of infected ants by herbivorous definitive hosts. The likelihood that an infected ant is eaten upon initial display of parasite-induced biting behavior may be very small. The chance of transmission of *D. dendriticum* will, however, increase with prolonged exposure to the definitive host and thus depend on ant host longevity. Thus, it is likely that natural selection favors increased exposure to the definitive host but disfavors exposure to pre-mature predation or hazardous environmental conditions, which could kill the ant host and, therefore, the parasite (Fritz 1982; Parker et al. 2009).

Studies dating back to the 1970s and 1980s reported anecdotal observations of *D. dendriticum-*infected ants biting in the morning and evening, leading to speculations on the possible role of time of day, temperature, or other environmental factors related to increased transmission success to grazing ruminant definitive hosts, but this was not investigated further (Tarry 1969; Badie et al. 1973; Badie 1975; Spindler et al. 1986; Badie and Rondelaud 1988). The chronobiology and effects of light have been shown to play a role in the expression of parasite-induced behavioral changes in other systems. In the case of *Ophiocordyceps unilateralis s.l.*-infected carpenter ants climb around solar noon and bite the vegetation in locations and orientations, which would lead to optimal humidity conditions for spore formation and spore release (Andersen et al. 2009; Hughes et al. 2011). Another host–parasite system similarly dependent on trophic transmission to *D. dendriticum* is the brain-encysting trematode *Microphallus papillorobustus* and acanthocephalans infecting gammarids. Infected gammarids seek light and cling to submerged macrophytes in the habitat of dabbling ducks which serve as definitive hosts (Bethel and Holmes 1973; Helluy 1983).

Our previous laboratory study indicated that the biting behavior of *D. dendriticum*-infected ants was determined by a negative relationship between increasing temperature and the probability that infected ants would bite a leaf (Botnevik et al. 2016).

However, the effect of temperature on *D. dendriticum* infected ant’s biting behavior has, so far, not been tested under natural conditions, where ants are exposed to multiple environmental variables with potential effects on ant behavior.

In this study, we recorded the manipulated phenotype of the second intermediate host, the European Red Wood Ant, *Formica polyctena*, naturally infected with *D. dendriticum*, concurrent with in situ measurements of temperature, relative humidity (RH), and time of day. In addition, we quantified the frequency of changes to and from the manipulated phenotype in relation to temperature on individually marked ants on 7 different days from August to October and at two separate sites.

We hypothesized that temperature was the most important factor in infected ants expressing the manipulated behavior in line with the previous laboratory study (Botnevik et al. 2016) and with circumstantial evidence from previous field observations (Badie et al. 1973; Paraschivescu and Raicev 1980; Spindler 1986). To our knowledge, this is the first study to individually label and track the behavior of infected ants in the field, which rendered the option to recatch individual ants and study their behavioral expression of infections over 24 h, and on longer term.

## MATERIALS AND METHODS

### Fieldsite and execution of fieldwork

Fieldwork took place in the Bidstrup Forests in Hvalsø, approximately 45 km Southwest of Copenhagen, Denmark, in 2016–2017. The forests contain a mixture of hardwood and coniferous trees in a hilly terrain and the *D. dendriticum-F. polyctena* system inhabits the forests (Botnevik et al. 2016).

The aims of the fieldwork were to 1) Investigate the relative contribution of temperature and RH (measured in the microclimate of infected ants), time of day, date, and an irradiation proxy in inducing and maintaining the altered phenotype (biting to vegetation). 2) Track biting behavior of individually marked infected ants to observe the dynamics of initiation, maintenance, and termination of the altered phenotype.

Four anthills were included in the observations each day (except in April 2017, see below) and were from two different sites (s and h). Three anthills; Anthill 1 (55°34ʹ41ʹʹN 11°52ʹ22ʹʹE), Anthill 2 (55°34ʹ40ʹʹN 11°52ʹ22ʹʹE) and Anthill 3 (55°34ʹ40ʹʹN 11°52ʹ23ʹʹE) were located in a line at approximately 1 m distance from each other (site s). Anthill 4 was located approximately 300 m from site s (55°34ʹ37ʹʹN 11°52ʹ43ʹʹE) and termed site h.

### Ant observations

Fieldwork was avoided on days with precipitation to standardize the conditions for ant behavioral studies. Ant observations were conducted from early morning to late afternoon/evening on nine days from 16 August 2016 until 12 October 2016, and on 3 days from 6 April 2017 to 16 August 2017. On one occasion, ant observations continued until sunset and continued the following morning, to cover a 24 h cycle (22 September 2016 to 23 September 2016). A total of 131.5 h of field observations were conducted from 12 occurrences, with one stretching 2 days, giving a total of 13 observation days from 16 August 2016 to 16 August 2017.

**Figure 2:**
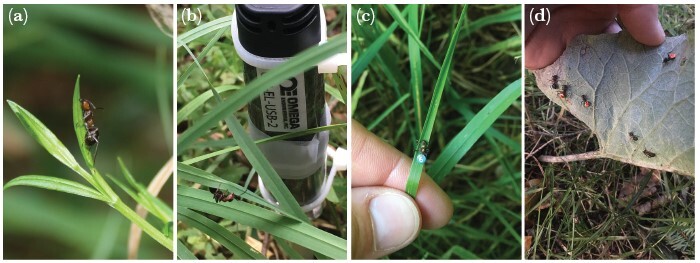
Photographs of *Formica polyctena* ants expressing the behavioral alteration induced by *Dicrocoelium dendriticum* in the Bidstrup Forests, Denmark, in 2016 and 2017. A, shows an ant expressing the biting behavior (on probably *Stellaria holostea*), and in B a temperature/humidity- logger is seen placed in close proximity to an ant expressing the altered behavior, biting onto cocksfoot grass, *Dactylis glomerata*. C with a number tag glued to the dorsal part of gaster (individual B6, also biting onto a piece of *Dactylis glomerata*). In D, an assembly of 6 infected ants were found under a big leave (undefined *Arctium* species), 4 of these with a number tag on (R33, R63, R76, and a B-tag not readable from this angle).

On each field day, the vegetation within a perimeter of 2 m from each anthill (3 m from Anthill 4 due to the dispersed vegetation at this site) was examined for ants biting to vegetation at a minimum of six times every day. A temperature/humidity -logger (OM-EL-USB-2, Omega Engineering Inc.) was attached with two plastic strips to a 1-m long marker stick and placed in the ground at the same height and in close proximity to the majority of ants biting to vegetation at the first observation time point (Figure 2B). Temperature and RH were recorded continually by the temperature/humidity-logger in intervals of 2 min. For the analysis, the median temperature and median RH were used for the minutes that did not have a recorded temperature and RH linked to it. For Anthill 2, the mean values from the surrounding anthills (1 and 3) were used for each timepoint. Five factors that potentially influenced the proportion of infected ants biting to vegetation were included in the analysis of ant activity: temperature, RH, date, time of day (day length divided in three equal groups of the total day length), and an irradiation-proxy (calculated from official records of sunrise and sunset, www.soltider.dk), indicating the level of solar irradiance that biting ants could be exposed to.

Thus, concurrent measures of temperature, RH, and the number of ants biting the vegetation were recorded at each of the minimally six observation time points per field day. In August–October 2016, 172 ants were individually marked (after the final observation time point of the day, of these 126 ants were observed again at the same anthills as they were at when tagged) by numbered and colored tags (Swienty A/S) glued to the dorsal part of gaster of the ants observed biting to vegetation (see Figure 2C,D, in-depth description of procedure in supplementary, data in dataset S3, and further analysis in the section “Persistence of biting behavior”).

Ants biting to vegetation received a touch stimulus, which induced slight movement in ants harboring *D. dendriticum* (Paraschivescu and Raicev 1980; Gasque and Fredensborg, personal observation) to distinguish them from infections by the entomopathogenic fungus *Pandora formicae* (de Bekker et al. 2018), which also inhabits the Bidstrup Forests (Małagocka et al. 2015, 2017). *P. formicae* initially induces a similar behavior, but infected ants remain motionless.

### Orientation of infected ants and infection patterns

On two separate days (15 September 2016 and 22 September September 2016, recorded, respectively in the afternoon and in the morning), the orientation of infected ants biting onto grass blades was noted. It was noted for each recording whether the ant was facing downwards toward the roots or facing upwards to the tip of the grass blade (see Figure 2C).

One hundred and seventy-four ants expressing biting behavior were collected on different dates (Supplementary Table S2) from the four observed anthills and transported back to the laboratory in individual 15 mL centrifuge tubes for dissection and verification of infection. Eighty ants collected randomly by the use of a handkerchief placed on the nest (Botnevik et al. 2016) were used as a negative control. Ants were stored at 5 °C until they were measured from mandible to the tip of gaster to the nearest 1 mm and dissected. Dissections were performed in a 0.9% saline solution under a Leica MZ12 stereomicroscope (×20), and the presence and number of metacercariae in the gaster were recorded (for the first two groups). For the last group collected on 16 August 2017, dissections were conducted only to verify the infection status of the ants.

### Statistical analysis

The number of ants biting onto the vegetation at the different observations were considered independent observations since the probability of expressing parasite-induced behavior was determined by the environmental factors at the time of observation (Supplementary Dataset S1). The number of ants expressing the altered phenotype fluctuated considerably between sampling days at a given anthill from a few individuals to 116. We, therefore, used the proportion of ants biting to vegetation (the number of ants biting at said time point relative to the maximum number of ants observed biting to vegetation at the same anthill that day) on each day as the dependent variable. Temperature, RH, date, an irradiation proxy, and time of day (interval) were used as explanatory variables.

We do not have an individual ID on the majority of the ants (only the 126 tagged ants mentioned earlier). Therefore, it was not possible to include individual ID as an independent variable in the analysis. With a proportional response variable, the data followed a binomial distribution, and we used logistic regression in a generalized linear model (GENMOD, SAS®) to assess the associations (see codes used in Supplementary Appendix 1).

Initially, all five explanatory variables were included in the model (Supplementary Table S1). By including or excluding date in the model, it was possible to assess the significance level of the other variables operating within and among dates, respectively. The significance levels of all the variables were compared between the two runs (including date: Supplementary Table S1 vs. excluding date: Table 1), to estimate which factor was the one explaining most of the expressed parasite-induced behavior of the ants within a day as well as across observation dates. By comparing the models with AIC, BIC, Deviance, Pearson chi-square, and more, the different tests pointed at the two different models of being the best-fitting model. So, to further analyze which factor had the biggest influence on the expression of the proportion of infection in our data set, we performed a regression analysis based on the Random Forest method in R (v 4.1.2). As the date itself was not regarded correctly, we instead included it in the format of year, month, and days per year as separate columns utilizing the R packages Dplyr (v 1.0.8) and Lubridate (v 1.8.0). The initially run included all the variables in Supplementary Dataset S1, besides the two used to calculate the proportion of infection (infection and maximum), as they obviously would skew the test. Lastly, excluding all the factors used to calculate irradiation proxy. We utilized the R package Boruta (v 8.0.0) to perform feature selection and ranking as a regression to predict the proportion of infection, based on 100 iterations of 20,000 trees. R package Caret (v 6.0-94) was utilized to split train and test datasets, and also train and test the models to predict the proportion of infection. Train and test splitting separated the data randomly, with 30% of the data retained as a testing set. We trained the model with 5-fold cross-validation repeated 10 times, utilizing forests with 2000 trees and 4 tuning length options. Model performance was evaluated with *R*^2^ and RMSE.

**Table 1:** Results of generalized linear model examining the effects of environmental factors and time on the expression of biting behavior in *Dicrocoelium dendriticum*-infected *Formica polyctena* given as the proportion of ants displaying the behavior at each observation in relation to the maximum number of ants observed displaying biting behavior on that day. “Irradiation-proxy” indicates the solar position on the sky and “Time of day” representing an interval as day length is divided into three groups.

Factor	Den df	Estimate	χ^2^	*P*-value
Temperature	437	−0.1090	120.94	<0.0001
Relative humidity	437	0.0145	26.17	<0.0001
Time of day	437	0.0544	0.50	0.4786
Irradiation-proxy	437	−0.0012	3.28	0.0703

The number of ants maintaining their attachment throughout the day (Supplementary Dataset S2) was used as a proportion to the maximum of ants at each anthill in relation to first; date, anthill, average RH and average temperature, and later solely average temperature and average RH at each anthill per day, in both cases, a generalized linear model with binomial errors (GENMOD) was chosen and tested in SAS^®^.

The orientation data of ants biting onto grass were analyzed by chi-square test compared to the equal chance of orientation to either direction.

Graphs were made in GraphPad Prism (v 9.5.1.), besides Supplementary Figure S1, which was generated in R and improved in Inkscape (v 1.2), and Supplementary Figure S2 was generated in Excel (v 2208).

## Results

### Factors determining parasite-induced behavior modification

Temperature (Den df = 437, χ^2^ = 120.94, *P* < 0.0001) was strongly associated with the proportion of infected ants expressing the manipulated phenotype (Table 1).

As seen from the estimates from the maximum likelihood parameters in the test excluding the date variable, temperature explains most of the variation in our dataset (Supplementary Dataset S1), as a 1 °C increase makes 11% of the ants let go of their biting (estimate −0.1090, SE ± 0.0106) and when including date (Supplementary Table S1) in the test the same accounts for 14% of the ants (estimate −0.1384, SE ± 0.0268). Both estimates are comparable as they fall within the same standard error. With the comparison between the date-included and date-excluded runs, temperature was the only variable of high importance in all tested models, which kept a high χ^2^ value, and highly significant *P*-value (*P* < 0.0001). In the excluded-date run, relative humidity (Den df = 437, χ^2^ = 26.17, *P* < 0.0001) was highly associated with the proportion of ants expressing the *D. dendriticum* infection. However, RH was not significantly related to ant-biting behavior within date, and the significant effect of RH observed across dates was likely an artifact of a negative correlation between RH and temperature with the latter being the main explanatory factor of ant-biting behavior (see above). Irradiation-proxy or time of day (by interval) showed no significant relationship to the induced behavioral modification in either of the runs. In a Random Forest regression modeled to predict the proportion of infection, the temperature was ranked as the most important factor for prediction (Supplementary Figure S1). The trained model could predict roughly half of the proportion of infection in the test set (*R*^2^ = 0.5297, RMSE = 0.2029), while a linear regression between the proportion of infection and temperature presented an *R*^2^ of ~0.25 in the full dataset.

### Persistence of biting behavior

**Figure 3:**
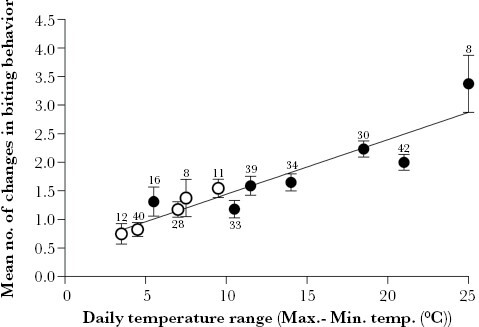
Number of changes in biting behavior (mean ± SEM) of individually marked *Formica polyctena* ants infected with *Dicrocoelium dendriticum* in relation to the observed temperature range (difference between maximum and minimum temperature) on seven (anthill 1-3, closed circle), or five observation days (anthill 4, open circle) between August and October 2016, in the Bidstrup Forests, Denmark (see Supplementary Dataset S3: “GasqueFredensborg_2023_S3_Individually_marked_ants”). The number of individually marked ants observed per site per day is indicated at each error bar. A significant positive relationship was observed between temperature range and the number of changes to the manipulated behavior for both sites (anthill 1-3, *R*^2^ = 0.77, anthill 4, *R*^2^ = 0.98, combined *R*^2^ = 0.86 (regression line: mean number of changes in behavior = 0.484 + (0.0957 × temperature range)), all *P* < 0.001).

The number of switches to and from biting behavior was strongly and positively related to the daily temperature range (difference between maximum and minimum temperatures) in individually marked infected ants across the observation days (Linear Regression: *F* = 61.1, df = 1, *P* < 0.001; Figure 3). Thus, the larger the difference between maximum and minimum temperature, the less persistent the manipulated biting behavior.

Labeled ants that were used in a 24-h study from 22 September 2016 to 23 September 2016 showed that of the numbered ants biting to vegetation in the evening (*N* = 30); 96.67% were observed in the same state at the same location the following morning.

### Seasonality

**Figure 4:**
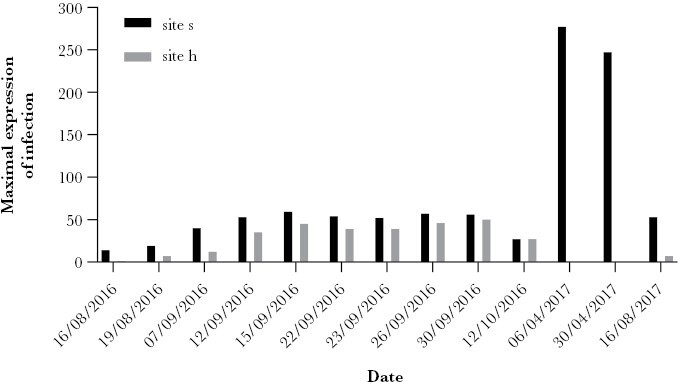
Bar chart representing the seasonal variation in the expression of *Dicrocoelium dendriticum*-induced biting behavior of *Formica polyctena* from two sampling sites (site s (anthill 1-3) and anthill 4, from site h) on 13 observation days (16 August 2016 to 16 August 2017) within 1 year at the Bidstrup Forests, Denmark. The bars represent the maximum counted number of ants expressing the manipulated state at any of the counting time points during each date as a total for both the sites (for site h: one number, for site s: maxima for anthill 1-3 added together) (see Supplementary Dataset S1: “GasqueFredensborg_2023_S1_Abiotic_factors”). In 2 days of April, at the h site, no ants were observed.

The number of biting ants counted on each observation day varied greatly (Supplementary Figure S2). However, a seasonal difference was observed from summer 2016 to summer 2017 when plotting the maximum number of infected ants counted on each day from the two sites (Figure 4). In April 2017, up to five times, the number of ants biting the vegetation were observed in comparison to the other recorded months at the same site.

### All-day attachment

**Figure 5:**
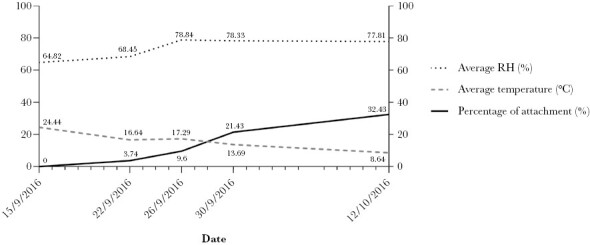
The percentage of maintained all-day attachment of *Dicrocoelium dendriticum* infected *Formica polyctena* ants (%, filled dark gray line) to the total of ants observed at all the anthills per day, throughout the observation time points of the assigned days (15 September 2016 to 12 October 2016), in relation to the average temperature (°Celsius) (dashed light gray line) and the average relative humidity (RH, %) (dotted line) measured in the microclimate of the ants that day (see Supplementary Dataset S2: “GasqueFredensborg_2023_S2_All-day_attachment”). The exact values of the three parameters for each day are given near the lines. On earlier dates (16 August 2016 to 15 September 2016), the percentage of all-day attachment was also 0%, as shown for 15 September 2016. (*N* total; 15/9: *N* = 166, 22/9: *N* = 187, 26/9: *N* = 177, 30/9: *N* = 168 and 12/10: *N* = 74).

At the end of September/start of October 2016, we observed that a great majority (up to 1/3) of the ants stayed in the biting state for the whole day of observations (Figure 5), in contrast to observations made earlier in the season (where none remained attached during all the observation times of the day). When analyzed, the date showed positive correlation to the proportion of the maintenance of biting (Num df = 4, χ^2^ = 20.26, *P* = 0.0004). Considering only the influence of the abiotic factors (average temperature and average RH for the individual anthills), average temperature showed the highest correlation to the maintenance of biting during the day (Num df = 1, χ^2^ = 17.05, *P* < 0.0001), whereas average RH showed no significant relationship to the proportion of ants biting to vegetation.

### Orientation of infected ants and infection patterns

None of the 80 ants randomly collected from the anthills were infected with *D. dendriticum* metacercaria (Supplementary Table S2). A majority of the ants (70%) expressing infection by biting on to vegetation were facing downwards (*n* = 144, df = 1, χ^2^ = 23.36, *P* < 0.0001). Combining all dissections, a metacercariae prevalence of 97% (*N* = 174) was found in ants collected when biting to the vegetation in the field.

## DISCUSSION

There is a need for studies on how parasitic manipulation of animal behavior responds to changes in environmental conditions (Loreto et al. 2018). However, it is inherently challenging to study the effects of individual environmental factors on animal behavior under natural conditions, as many of them cannot be controlled and/or may co-vary. In this study, we succeeded to monitor the biting behavior of the ant host in relation to individual environmental factors under natural conditions. We usually observed the greatest numbers of ants in the biting state in the early morning and in the late afternoon/evening, similar to previous studies (Supplementary Figure S2) (Badie et al. 1973; Badie 1975; Spindler 1986; Badie and Rondelaud 1988; Manga-González et al. 2001). However, our analysis clearly demonstrated that temperature, not the time of day itself, explained the switch to a biting state observed in the manipulated phenotype (biting to vegetation). Thus, low temperature facilitated the biting behavior while higher temperatures reduced it (Table 1; Figures 3 and 5). We also found a significant positive relationship between the daily temperature range and the number of times infected ants switched biting behavior between the manipulated and non-manipulated phenotype (Figure 3). This indicates that the persistence of the biting behavior was greatly reduced when ants experienced large variations in the temperatures during a day. The results from this field study therefore corroborate with results from a previous laboratory-based study, in which temperature determined the probability that infected ants were found biting to grass leaves (Botnevik et al. 2016).

In further support of temperature as the main driver of the induced biting behavior, the proportion of infected ants maintaining all-day attachment significantly increased from the middle of September until the middle of October concurrent with a decrease in daily average temperature (Figure 5). Thus, in October, infected ants were more likely to remain attached to the vegetation the entire day when the maximum temperature was 11.5 °C. Termination of the parasite-induced behavior, therefore, seems to be determined by temperature, and not by time of day, RH, or a proxy for irradiation. Similarly, as indicated by our 24-h study, ants presumably remain attached all night. It is from late evening to early morning that we measured the lowest temperatures. In April 2017, there were up to five times as many ants observed exhibiting the manipulated phenotype than in other months (Figure 4), suggesting that infected ants survive the winter in the nest and continue to express the manipulated behavior the following spring as transmission between snail and ant host primarily takes place during late spring and summer (Tarry 1969; Manga-González and González-Lanza 2005). Interestingly, late May to early June is when female roe deer express the highest total daily activity time (56.7% of the day, Cederlund 1989) in central Sweden (similar latitude to our study). The largest proportion of infected ants biting may therefore, not only coincide with the daily activity of potential definitive hosts (Stache et al. 2012) through the indication by temperature but potentially also be synchronized with the seasonal activity of potential other definitive hosts (Cederlund 1989). Temperature seems to be an appropriate indicator of parasite transmission success and, thus, parasite fitness, as it relates to both host longevity and the encounter possibility with potential definitive hosts. Hence, biting to vegetation could be lethal to ants and, thereby the *D. dendriticum* metacercariae at peak temperatures. In addition to host mortality, temperature also provides an indicator for the most opportune daily transmission window to potential definitive hosts since low temperature coincides with the crepuscular activity of herbivorous definitive hosts, for example, the European roe deer (Stache et al. 2012).

The exact mechanism linking temperature to climbing and biting behavior of *D. dendriticum*-infected ants remains unknown. However, it is known that one or few metacercariae lodge at the suboesophagal ganglion in the ant host (Romig et al. 1980; Mart**í**n-Vega et al. 2018, see arrow in Figure 1), which controls the initiation of mandibular abductor and adductor movement (Chapman 2013). Changes in temperature might affect parasite or host secretion of molecules, an inflammatory response or the mechanical pressure of the parasite on mandibular nervous tissue may in turn provoke a biting behavior at low-temperature conditions. Penetration of muscles by the fungus *O. kimflemingiae* may lead to the hypercontractions seen in the mandibles of infected carpenter ants (Mangold et al. 2019). However, in contrast to behavior-manipulating trematodes and viruses (termed neuroparasites; Hughes and Liebersat 2018), hypocrealean fungi do not invade the CNS of the living host (Fredericksen et al. 2017), whereas recent evidence suggests that entomophthorelean species can infiltrate the CNS tissue while the host is still alive (Elya et al. 2018, 2023). This all suggests that similar behaviors may be regulated by different underlying mechanisms across parasite phyla (Mangold and Hughes 2021).

In accordance with previous studies, we also found that most of the infected ants were facing head downwards (Badie and Rondelaud 1988; Manga-González et al. 2001). The reason is unknown, although we wonder if the final orientation is the result of a method for seeking out the optimal conditions of the attachment for the trematode-host or to provide shading at least for the head part, where the SOG-attached metacercaria is located (Gullan and Cranston 2010).

Our analysis indicates that the effect of RH on *D. dendriticum*-infected ants expressing biting behavior in the field is smaller than that of temperature in line with previous studies, conducted under controlled laboratory conditions where no interaction between relative humidity and biting behavior could be detected (Botnevik et al. 2016).

We indirectly assessed the effect of solar irradiation, by including a proxy for solar position in the analysis, on the biting behavior of ants infected with *D. dendriticum*. We did not find any evidence that the solar position had an effect on biting behavior, similar to a study on the effect of artificial light (Botnevik et al. 2016). Light plays an important role in other systems, where parasites initially induce the same attachment to foliage, such as fungi from the genera *Pandora* and *Ophiocordyceps. O. unilateralis s.l.,* makes the infected *Camponotus leonardi* ants attach approximately 25 cm above the forest floor, which should be the optimal microclimate for the post-mortem development of the stalk and subsequent spore release (Andersen et al. 2009; Hughes et al. 2011). Transition to the so-called death grip in naturally infected *Camponotus* ants is synchronized to solar noon in the field (Hughes et al. 2011; Will et al. 2020), and comparisons between *O. camponoti-atricipis-*infected *Camponoti atricipis* ants in shaded and control areas indicated a strong positive phototactic influence (Andriolli et al. 2019). These results combined suggest that illumination is an essential cue for fungus-infected ants to locate the optimal microclimate for progeny development and dispersal (Chung et al. 2017). Light has a pivotal role, not only in fungal systems but also for the expression of other parasite-induced behavioral alterations. Studies on aquatic gammarids infected with larval trematodes or acanthocephalans, demonstrated positive phototaxis of infected individuals, presumably increasing predation of infected gammarids by duck final hosts (Bethel and Holmes 1973; Helluy 1983). In that system, temperature had no effect on parasite-induced host behavior manipulation since light and not temperature presumably determined the encounter possibility with the definitive host (Labaude et al. 2020). For both of the baculoviral-induced phenotypic changes (hyperactivity and tree-top disease) the expressions are influenced by light (Kamita et al. 2005; van Houte et al. 2014; Bhattarai et al. 2018). Therefore, in future field studies, it would be relevant to test the effect of light intensity on the timing of behavioral alteration in ants infected with *D. dendriticum*, including a broader range of seasons.

For most host–parasite systems, the underlying mechanisms of induced parasitic manipulation, are to a great extend unknown. The same accounts for the *D. dendriticum-Formica* ant host sytem. By comparing similarly expressed behavioral manipulations across phyla, we speculate that the parasite-encoded protein tyrosine phosphatase (PTP) could be a potential regulating factor in the *D. dendriticum* case. PTP is linked to a change of movement and positive phototaxis, which is induced in hosts by several manipulating parasites. This enzyme has been found to play a role in the induction of hyperactivity in caterpillars when infected with baculoviruses (Kamita et al. 2005; Katsuma et al. 2012; van Houte et al. 2012). Fungal-encoded PTP is also upregulated during the manipulated state (deathgrip) of *Ophidiocordyceps*-infected ants (de Bekker et al. 2015, 2017; Will et al. 2020). Since the initial part of the *Ophiocordyceps-* and *Dicrocoelium-*induced behaviors in the ant host (the at minimum negative geotaxism (or positive phototaxis in *Ophiocordyceps* case) and the attachment to the vegetation by mouthparts) are similar to each other, PTP could have evolved convergently (Will et al. 2020). Currently, it is unknown whether the phenotypical similarities of infected host behavior have evolved independently across a taxonomically diverse range of parasites, or if they have evolved to exploit the same ancient host trait of biting to vegetation while sleeping (Lovett et al. 2020). In any case, investigating the possible role of PTP and other actors in the manipulated state of ants infected with *Dicrocoelium* would be very interesting to include in future quantitative studies such as transcriptomic or proteomic approaches (Biron et al. 2005, 2006; Małagocka et al. 2015; de Bekker et al. 2017).

## CONCLUSION

This study provided rare field evidence on the effects of environmental factors on parasite-induced behavioral changes in the host. We found that temperature was the driving factor influencing parasite-induced ant-biting behavior in the field. We propose that temperature sensitivity is an adaptation to increase transmission to the definitive mammalian host, while at the same time protecting the intermediate host from exposure to lethal temperatures. Investigating the biochemical underlying mechanisms and comparing these to other parasites inducing similar behavior in ants and other insects will be an important next step.

## Supplementary Material

arad064_suppl_Supplementary_Materials

## Data Availability

Analyses reported in this article can be reproduced using the data provided by Gasque SN, Fredensborg BL. 2023a (Dataset S1, Abiotic factors), Gasque SN, Fredensborg BL. 2023b (Dataset S2, All-day attachment) and Gasque SN, Fredensborg BL. 2023c (Dataset S3, Individually_marked_ants).

## Notes

We thank Per Moestrup Jensen for fruitful theoretical discussions and for statistical assistance, the management at Bidstrup Forests for access to fieldsites, Pedro Beschoren da Costa for cross-validation of GLM test results by application of the Random Forest method and Vera I. D. Ros and Julia Friman for comments on earlier drafts of the manuscript. Lastly, we also thank two anonymous reviewers whose constructive criticism improved the quality of the manuscript.
