# Supplementary figures and images for: Expression of trematode-induced zombie-ant behavior is strongly associated with temperature
Source: Behav Ecol. 2023 Aug 24;34(6):960–8. doi: 10.1093/beheco/arad064 (PMC10636736; doi:10.1093/beheco/arad064)

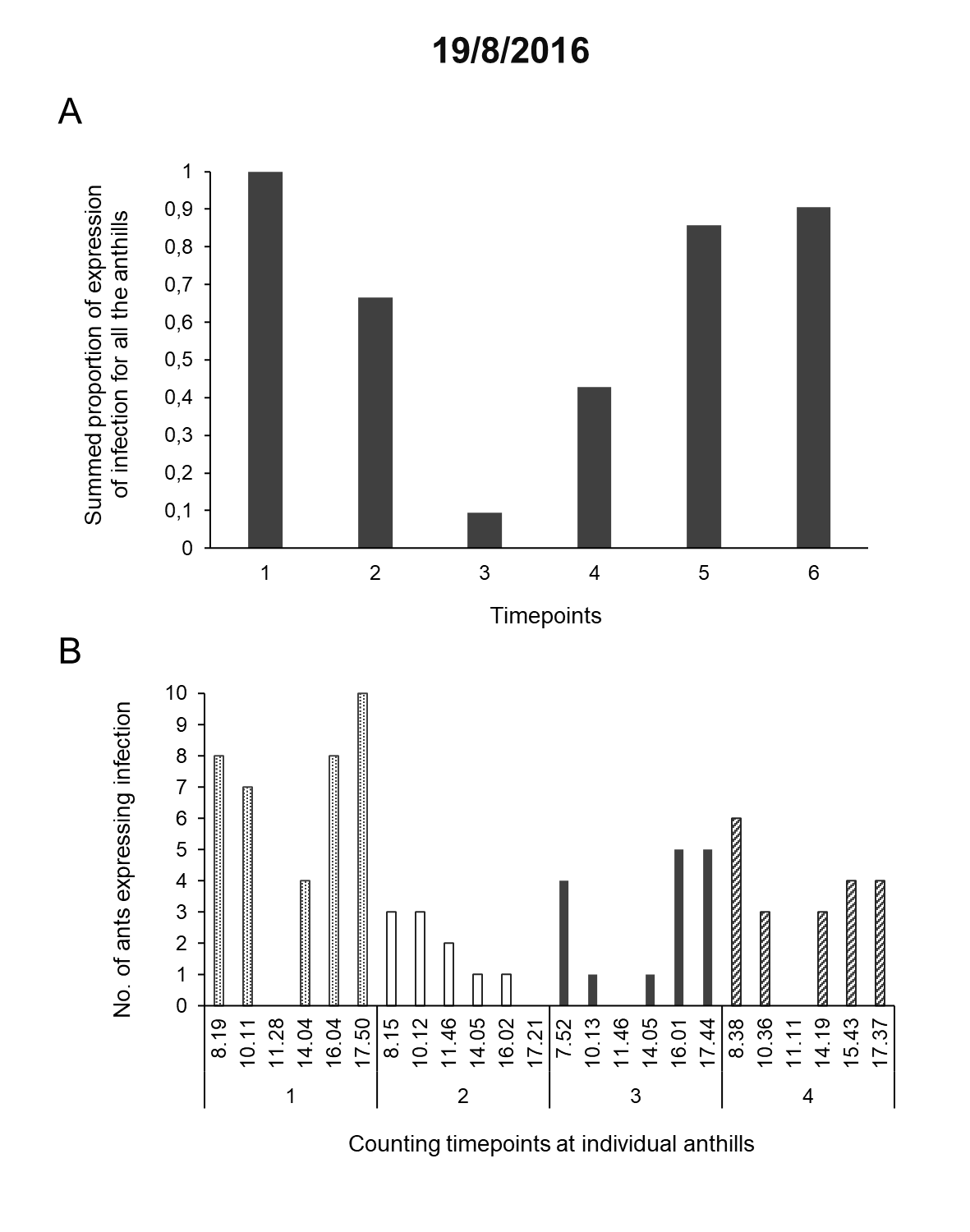

Supplement: arad064_suppl_Supplementary_Materials [file arad064_suppl_supplementary_materials.zip › Fig S2_Slide1.TIF]

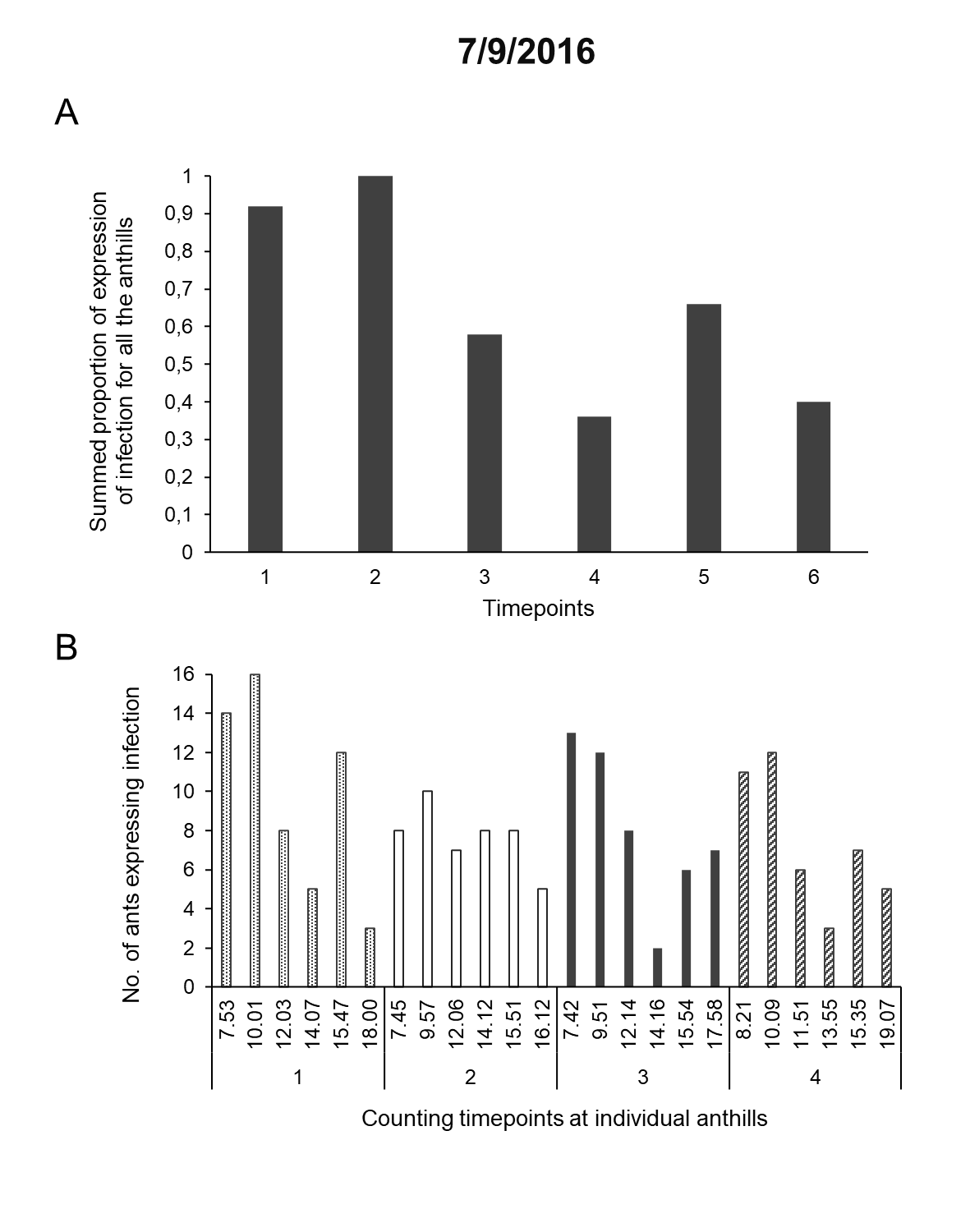

Supplement: arad064_suppl_Supplementary_Materials [file arad064_suppl_supplementary_materials.zip › Fig S2_Slide2.TIF]

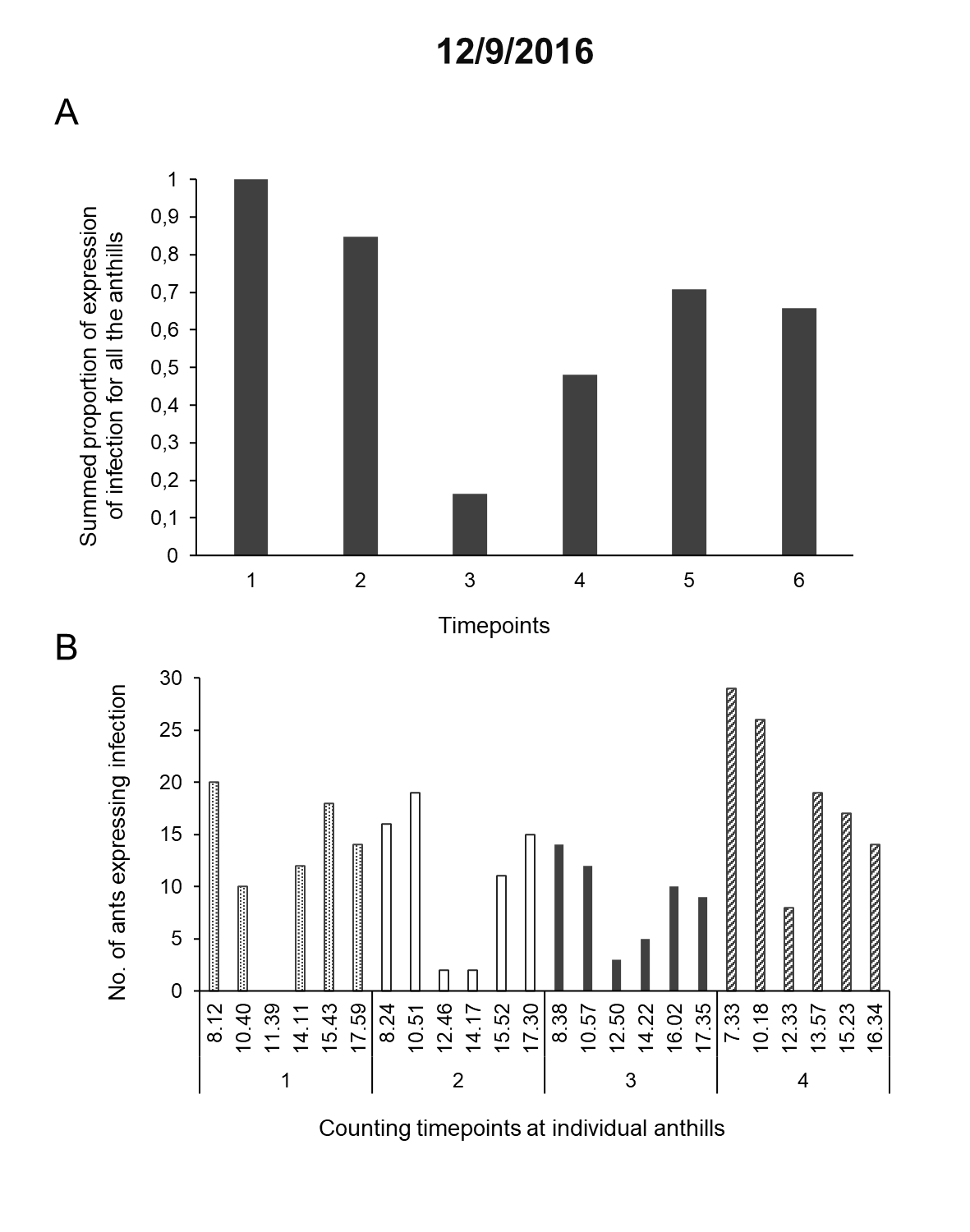

Supplement: arad064_suppl_Supplementary_Materials [file arad064_suppl_supplementary_materials.zip › Fig S2_Slide3.TIF]

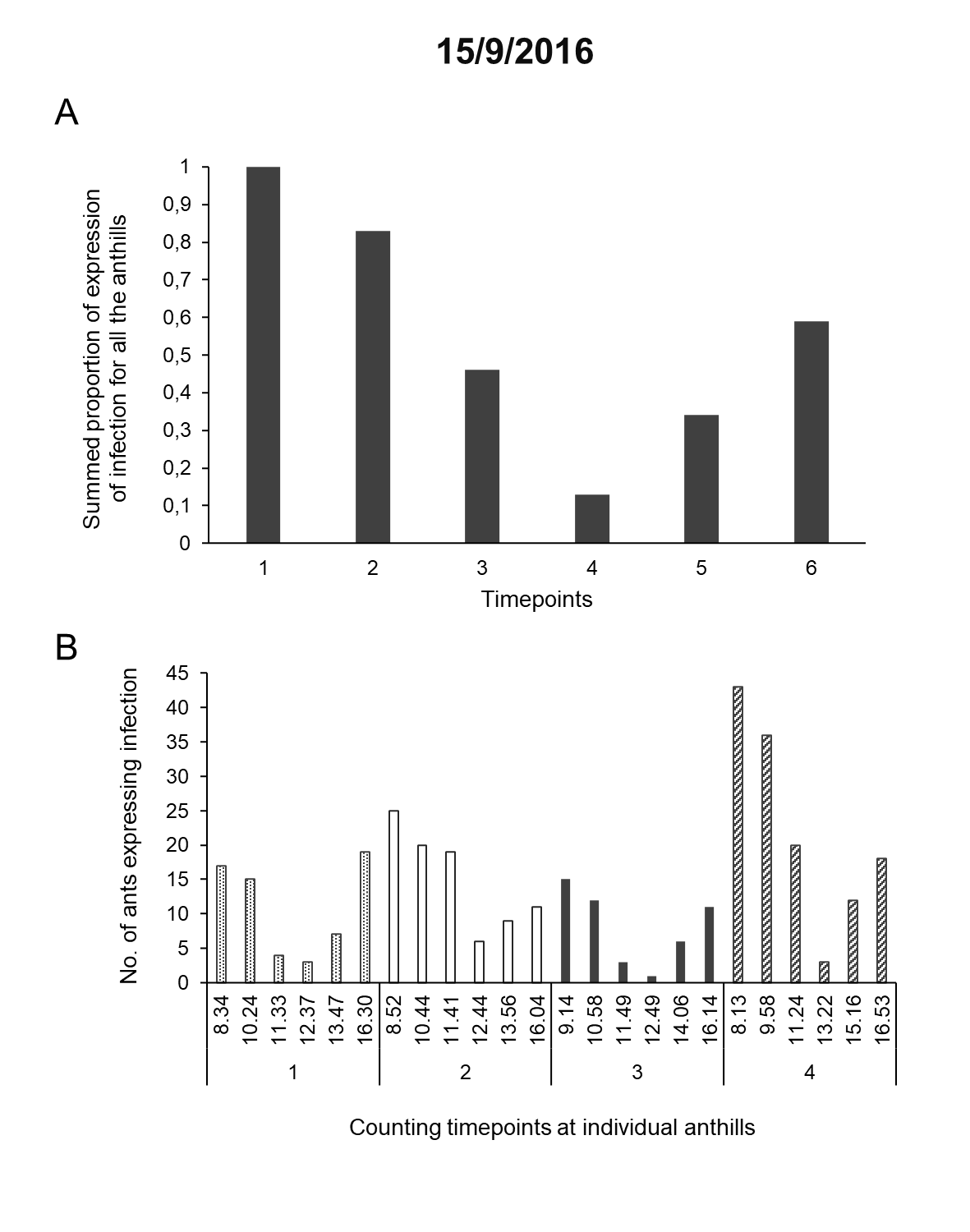

Supplement: arad064_suppl_Supplementary_Materials [file arad064_suppl_supplementary_materials.zip › Fig S2_Slide4.TIF]

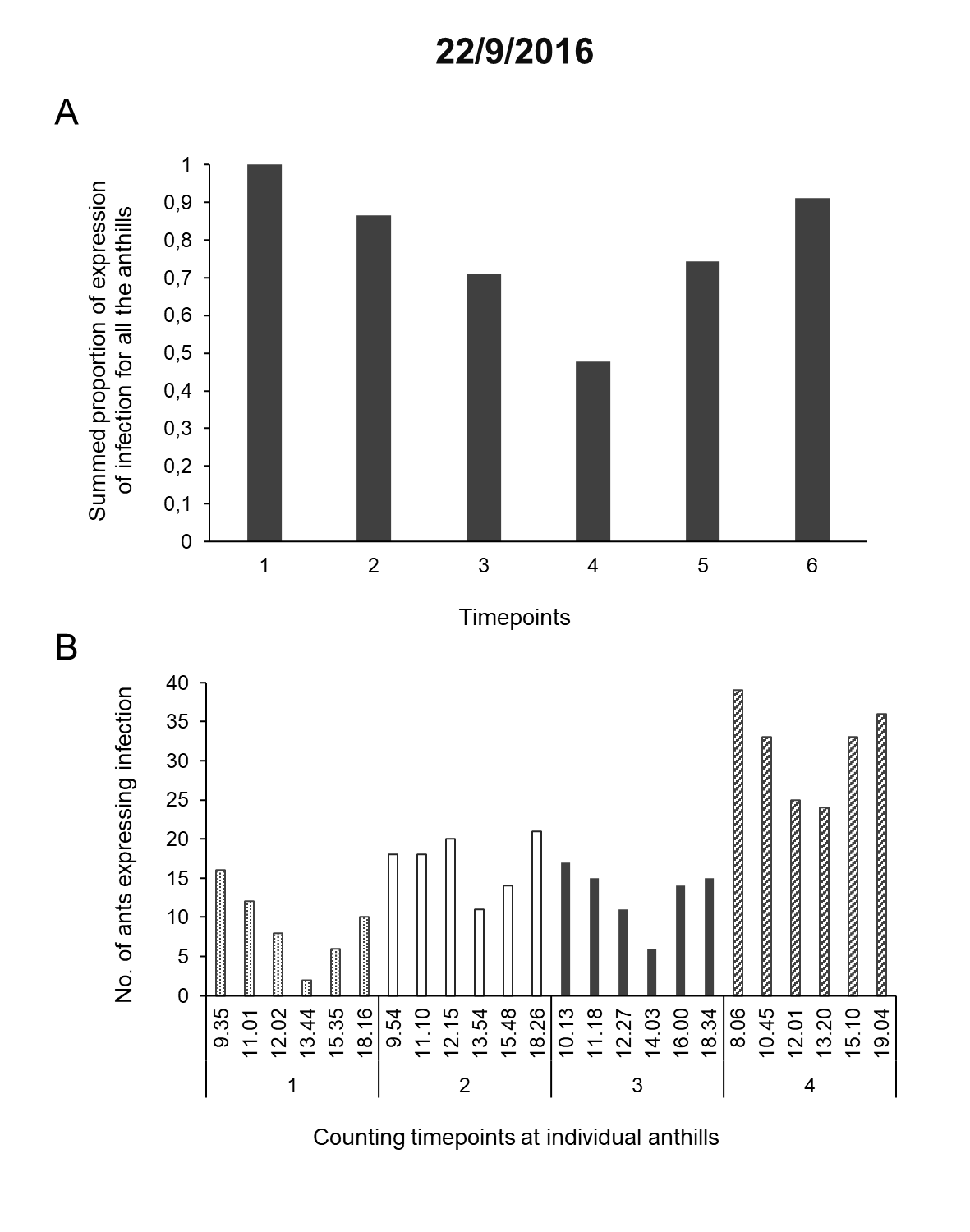

Supplement: arad064_suppl_Supplementary_Materials [file arad064_suppl_supplementary_materials.zip › Fig S2_Slide5.TIF]

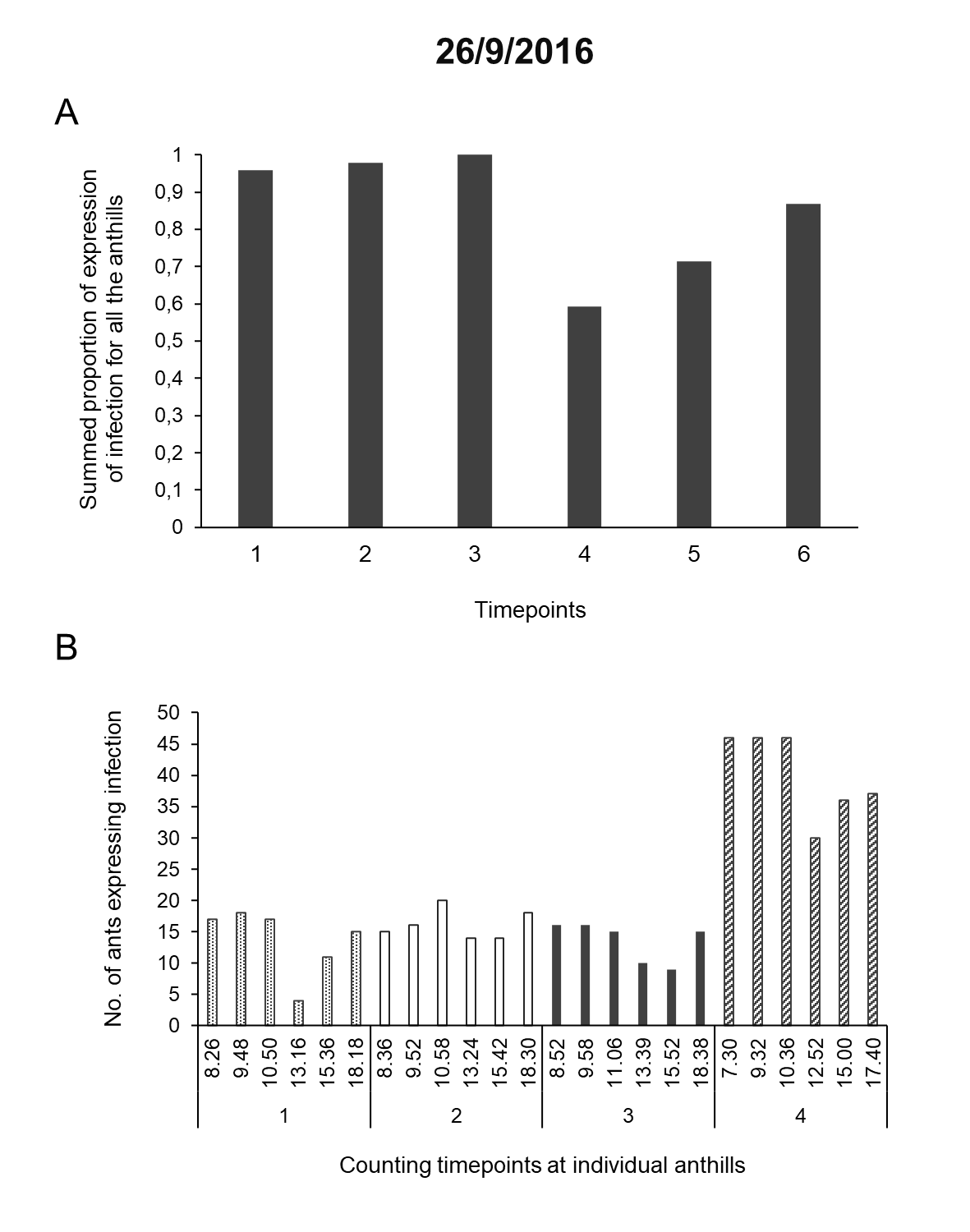

Supplement: arad064_suppl_Supplementary_Materials [file arad064_suppl_supplementary_materials.zip › Fig S2_Slide6.TIF]

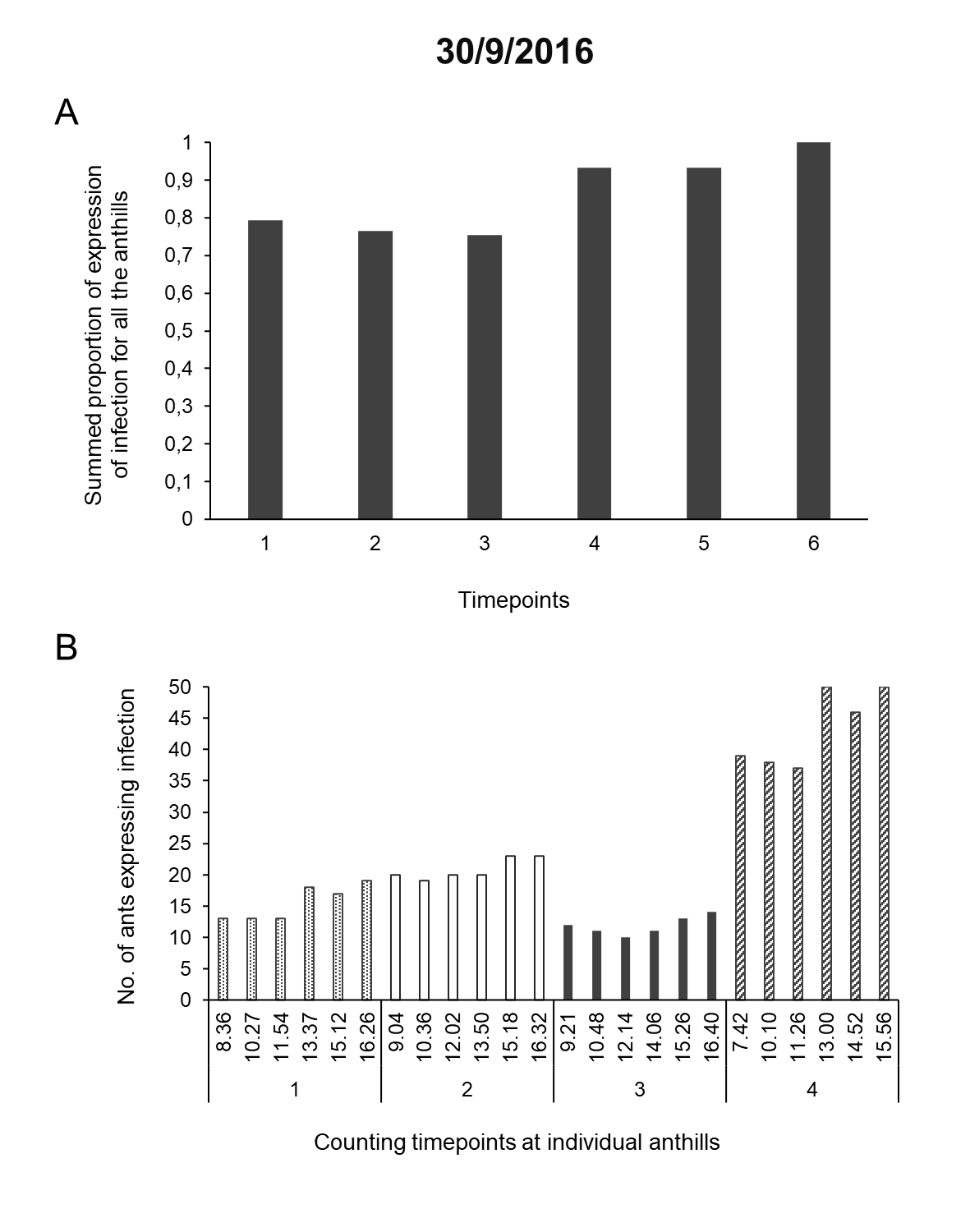

Supplement: arad064_suppl_Supplementary_Materials [file arad064_suppl_supplementary_materials.zip › Fig S2_Slide7.TIF]

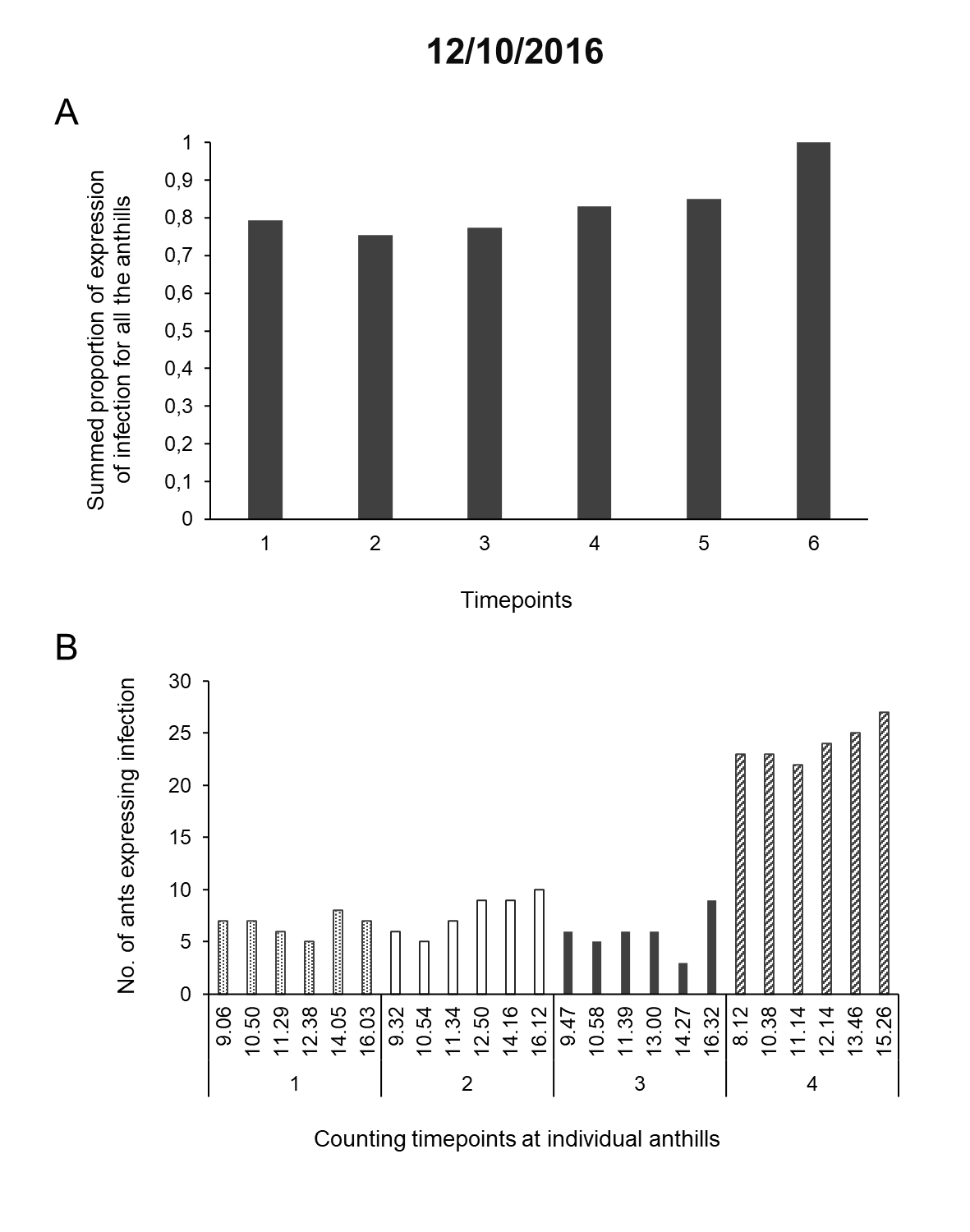

Supplement: arad064_suppl_Supplementary_Materials [file arad064_suppl_supplementary_materials.zip › Fig S2_Slide8.TIF]

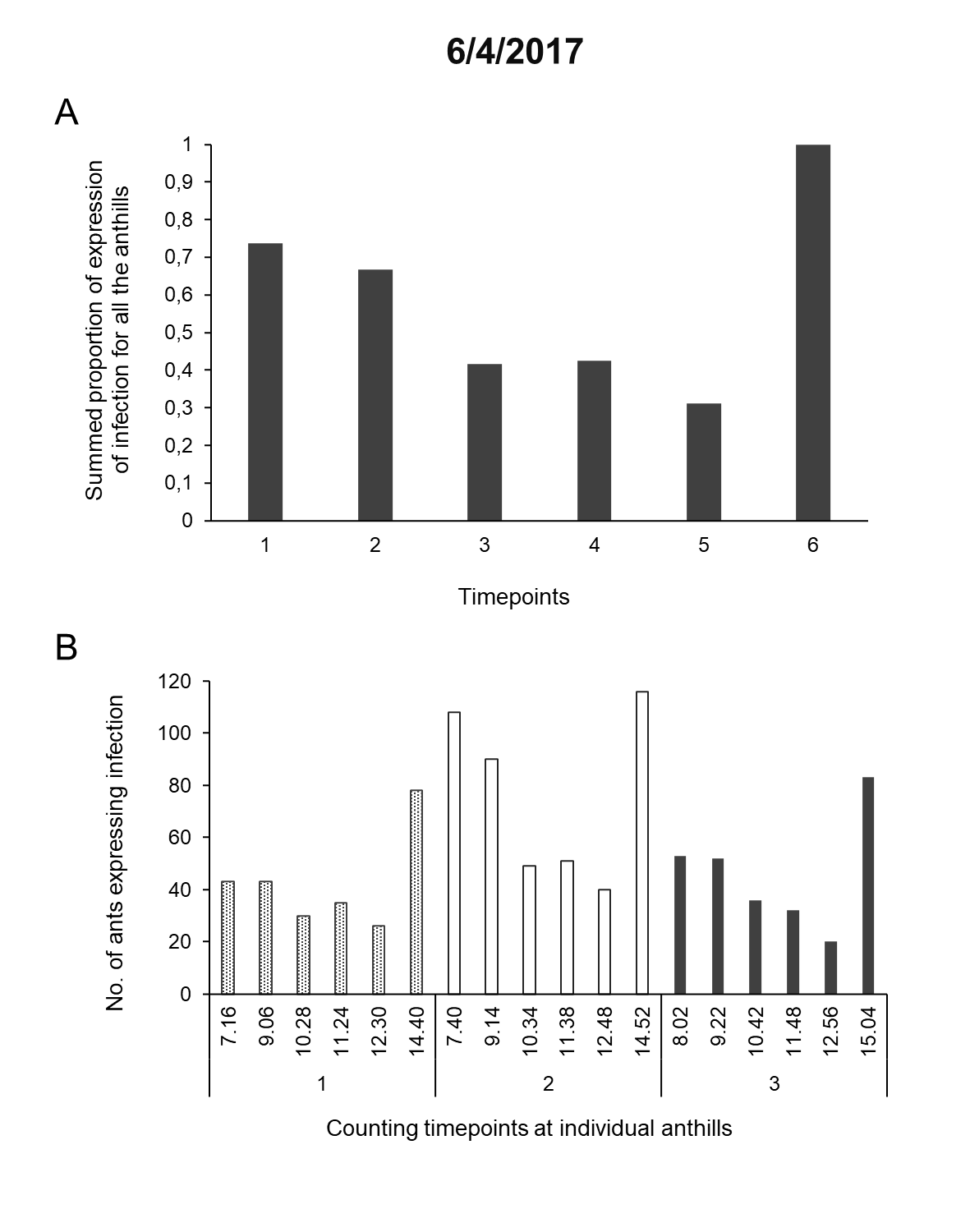

Supplement: arad064_suppl_Supplementary_Materials [file arad064_suppl_supplementary_materials.zip › Fig S2_Slide9.TIF]

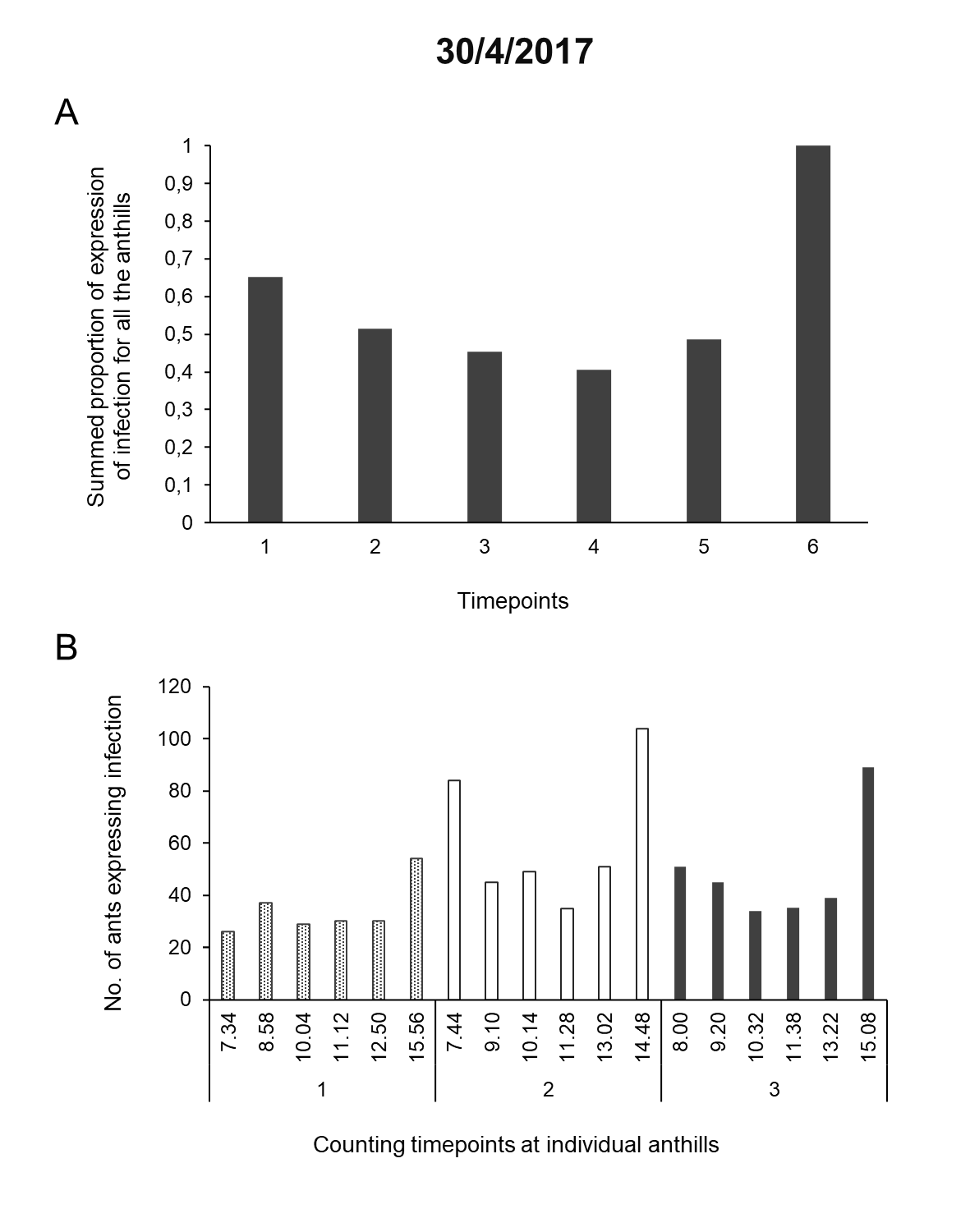

Supplement: arad064_suppl_Supplementary_Materials [file arad064_suppl_supplementary_materials.zip › Fig S2_Slide10.TIF]

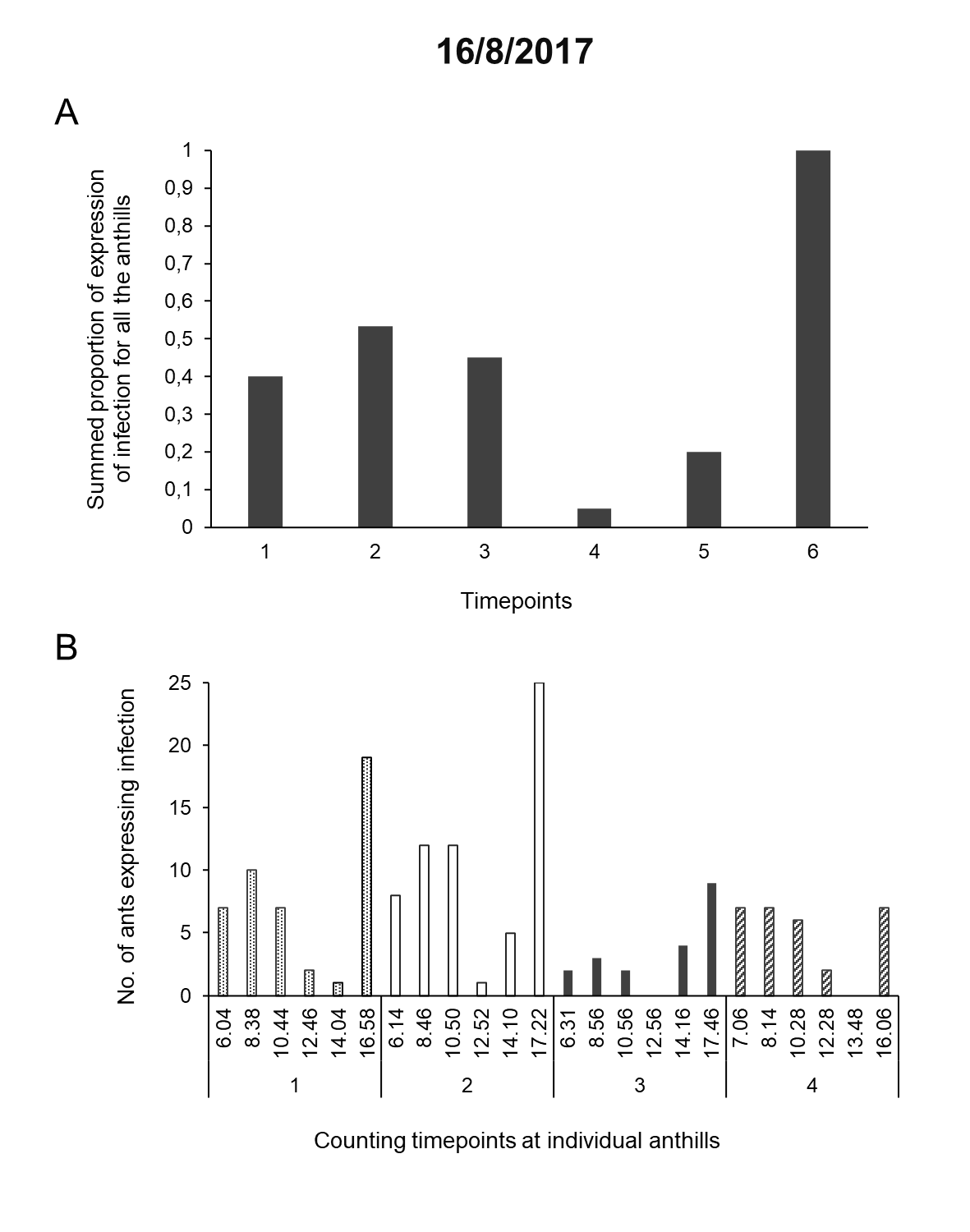

Supplement: arad064_suppl_Supplementary_Materials [file arad064_suppl_supplementary_materials.zip › Fig S2_Slide11.TIF]
